# Design and Implementation of a Wireless Sensor Network for Seismic Monitoring of Buildings

**DOI:** 10.3390/s21113875

**Published:** 2021-06-04

**Authors:** Julio Antonio Jornet-Monteverde, Juan José Galiana-Merino, Juan Luis Soler-Llorens

**Affiliations:** 1 Department of Physics, Systems Engineering and Signal Theory, University of Alicante, Crta. San Vicente del Raspeig, s/n, 03080 San Vicente del Raspeig, Spain; julio.jornet@ua.es; 2 University Institute of Physics Applied to Sciences and Technologies, University of Alicante, Crta. San Vicente del Raspeig, s/n, 03080 San Vicente del Raspeig, Spain; 3 Department of Earth Sciences and Environment, University of Alicante, Crta. San Vicente del Raspeig, s/n, 03080 San Vicente del Raspeig, Spain; jl.soler@ua.es

**Keywords:** wireless sensor networks, Wi-Fi networks, CC3200, Node.js, ambient vibrations, data acquisition, building monitoring

## Abstract

This article presents a new wireless seismic sensor network system, especially design for building monitoring. The designed prototype allows remote control, and remote and real-time monitoring of the recorded signals by any internet browser. The system is formed by several Nodes (based on the CC3200 microcontroller of Texas Instruments), which are in charge of digitizing the ambient vibrations registered by three-component seismic sensors and transmitting them to a central server. This server records all the received signals, but also allows their real-time visualization in several remote client browsers thanks to the JavaScript’s Node.js technology. The data transmission uses not only Wi-Fi technology, but also the existing network resources that nowadays can be found usually in any official or residential building (lowering deployment costs). A data synchronization scheme was also implemented to correct the time differences between the Nodes, but also the long-term drifts found in the internal clock of the microcontrollers (improving the quality of records). The completed system is a low-cost, open-hardware and open-software design. The prototype was tested in a real building, recording ambient vibrations in several floors and observing the differences due to the building structure.

## 1. Introduction

The degree of building damage caused by earthquakes is strongly related to the soil characteristics (local site effects) and the dynamic behavior of the structures [1]. In the case of the structures, numerical modeling and experimental measurements are widely used for estimating the dynamic properties of a building, although only experimental procedures allow obtaining the real behavior.

Earthquakes [2,3,4,5], forced vibrations [6,7] and ambient vibrations [8,9,10] can be used as input signals. Earthquakes and forced vibration recordings are costly options that can be applied in a reduced number of selected buildings. Besides, in the case of the earthquakes, permanently monitored buildings are also required. Thus, ambient vibration (or ambient noise) measurements have become a widely used alternative, as it is the fastest, cheapest and easy-to-implement experimental approach. In this case, a minimum of two three-component accelerometers or seismometers located on the ground and top floors can be used [11], although more extensive measurements are usually implemented [12]. Regarding the recording duration, measurements ranging from a few minutes [1] to several days [13] can be found in the literature. Once the data is recorded, it is subsequently processed to estimate the building properties, such as the fundamental frequency [14,15]. Some of these techniques are the horizontal-to-vertical spectral ratio (H/V or HVSR) [16,17,18] and the standard spectral ratio (SSR) [19]. In the case of the H/V method, the spectral ratio between the average of the two horizontal components and the vertical component, measured on the highest level of the structure, provides an estimation of the resonant frequency of the monitored building structure [11,20]. The fundamental frequency can be also estimated by the SSR technique, calculating the spectral ratio of the horizontal components (longitudinal and transversal) registered on the top floor and the same components registered on the ground floor [21].

In this context, that is ambient noise monitoring in buildings, most of the time the recording of the signals is carried out in situ, with seismographs that record the signal in the internal memory. Once the measurements have been completed, the data are recovered individually from each of the seismographs used, either by connecting a computer to them or by copying the memory cards one by one [1,11,22].

Another alternative is to wire the different sensors to multichannel data acquisition equipment, which in turn can be connected to a computer [12]. One of the limitations of these systems is that they use cabling to interconnect the different sensors with the recorder and this can severely limit the height of the building to be monitored. Another limitation is the impact of the signal-to-noise ratio, SNR, as the length of the cabling increases and also the interference that can be introduced in the recorded signal.

There are wireless seismographs on the market that do not have the limitations mentioned above but are very expensive, such as Unite System by Sercel [23], Sigma Systems by iSeis [24], or FierFly System by INOVA [25]. We can also mention the one developed by Jilin University [26].

The high cost of instrumentation and the rise of low-cost microcontrollers, each time with more functionalities, is what motivates the increasing number of proposals for measurement systems developed by the research groups themselves. Thus, wireless solutions can be found in the literature for seismic exploration [27,28], microzonation studies [29,30,31,32,33,34] and building monitoring [35,36,37], among others.

In the case of building monitoring, which is the research framework of this work, Nastase et al. [35], proposed a mixed system with wired and wireless connections. In this work, each sensor is wired to an analog-to-digital converter and then into a data acquisition computer. All these computers are connected to one router, which was in turn connected to a wireless base station that communicated with the computer systems outside of the building. A GPS Network Time Server with an antenna placed on the building’s balcony was used for synchronization.

In the work of Hou et al. [36], the proposed system consists of a base station, a computer and several wireless nodes connected in star topology. Each node includes four main units: flash storage, wireless transmission, microprocessing, and power management. The wireless transmission is carried out by the Texas Instruments CC2520 and CC2951 modules, using the ZigBee/IEEE802.15.4 protocol. They are used for sending and receiving orders and data. However, no consideration is found regarding data synchronization, which is very important when several sensors are monitoring simultaneously.

Finally, Valenti et al. [37] developed a system formed by two wireless sensor nodes connected to a wireless sensor network concentrator, which is in charge of sending commands, maintaining the synchronization, and identifying any malfunctions. In this case, the data are stored in a local memory and transmitted after the acquisition ends, using a polling scheme.

For short measurement periods, below one hour, it is important that all sensors are initially synchronized. However, for longer periods, in the order of several days, it is important that the synchronization is maintained throughout the entire measurement period, avoiding possible drifts of the internal clocks. In addition, in these cases, it is very important to have remote and real-time monitoring of the data, what allows verifying, at any time and from anywhere, that the recording is being carried out correctly.

Therefore, one of the great challenges in distributed acquisition systems is the perfect synchronization of the different Nodes, since without this feature the acquired signal tends to move and produces errors in the calculations of different parameters such as propagation speed, positioning of the origin of the movement, etc. One of the disadvantages of the existing microcontroller boards in the market is that they use a poor-quality quartz clock to lower their prices. These clocks are not very accurate and produce deviations that in prolonged recordings cause the time differences to be even greater and can be clearly perceived. For this reason, a great deal of effort has been devoted to implementing algorithms capable of synchronizing the Nodes. A starting point for the study of existing wireless sensor network (WSN) algorithms and protocols is presented by Sundararaman et al. [38], where different techniques are summarized and compared.

The basis of this work is an adaptation of the scheme proposed by Jornet-Monteverde and Galiana-Merino for multi-zone air conditioning systems [39] and an evolution of the works of Soler-Llorens et al. for wired [40] and wireless [31] seismic noise acquisition systems. In these last works, the samples are stored locally for later recovery and post-processing. In the wireless prototype [31], Zigbee technology is used to set up and control the registering process, although there is no real-time information on the recorded data. In the wired prototype [40], the registered signal can be monitored in real-time if a laptop is connected to the system. Therefore, in none of these works the signal can be stored and monitored remotely.

In this sense, the designed prototype gathers in a single system all the characteristics mentioned above. It provides remote and real-time control, data monitoring and saving, as well as data synchronization along all of the measurement period. For that, the proposed system uses not only the wireless communication, but also the existing network resources of the monitored building, reducing the expenses. Finally, the implementation is carried out with low-cost microcontrollers and microcomputers, providing an open-hardware and open-software system.

For a correct choice of protocol and algorithm it is necessary to take into account the topology of our WSN. In our case we propose a point-to-multipoint topology, with a server that provides the clock reference to the different Nodes under a wired and wireless network infrastructure. In this work we developed a wireless seismic acquisition system capable of displaying the sensor signal in real time and at low cost. Wi-Fi technology is used to provide the Nodes with access to the internal network of the building to be monitored and through this network the messages containing the signal samples are transmitted to the server, which can be located anywhere as long as it is connected to the same network. According to the Spanish Institute of Statistics, 91.4% of households had internet access and almost all of them, 99.7% (15 million households), had broadband internet in 2019 [41]. In United States, more than 80% of households had also internet in 2016 [42]. Thus, the use of the internal network connections in the monitored building is widely justified.

It is important to emphasize that each Node must be perfectly synchronized with the server, so the local time is also obtained. The proposed system does not use wiring and each Node is an independent entity that is controlled only by the server.

The main novelties of the designed system can be summarized in these points: (1) the data interconnection scheme between Nodes and Server uses not only wireless communication, but also the existing network resources that nowadays can be found usually in any official or residential building (lowering deployment costs); (2) the implemented data synchronization approach takes into account not only the time differences between the Nodes, but also the long-term drifts found in the internal clock of the microcontrollers, what improves the quality of records; (3) the system offers remote control access, but also remote and real-time monitoring and saving of the measured signals; (4) it is based on low-cost instrumentation. Another advantage is that the system is totally modular and up to 38 Nodes can be configured in theory, depending on the internal network traffic. In our case, the system was tested with up to two Nodes, recording ambient vibrations in a two-floor house.

## 2. Materials and Methods

### 2.1. Model Description

In order to monitor and record the vibrations that occur in different zones of a building in real time, a model based on the Client-Server concept was designed. The clients are implemented using a Texas Instruments (TI) CC3200 microcontroller [43] (Nodes) and the server is configured though a Raspberry Pi computer [44] (RPI Server). The system consists of several Nodes located on different floors of the building. Each Node incorporates a specially designed expansion board with a conditioning circuit [40] (page 5), which is connected to a three-component seismic sensor for the X-Y-Z axes. The Nodes are connected to the Wi-Fi on each floor where they are located and through the building’s internal local network they connect to the RPI Server via a TCP (transmission control protocol) connection. A JavaScript (JS) server was developed in the RPI Server that manages the TCP connections of the Nodes and receives the samples from each Node. Besides, the received data are saved in local files and displayed through a web user-interface.

**Figure 1:**
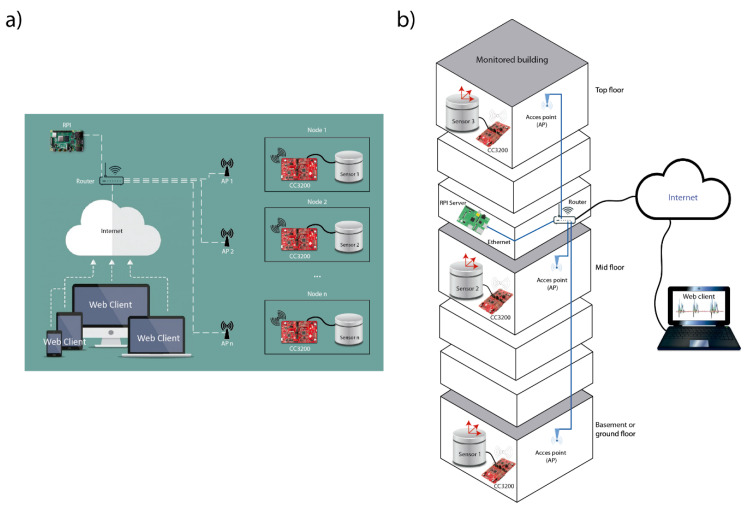
(**a**) General scheme of the developed system and its elements. (**b**) General three-dimensional schematic of a monitored building.

Figure 1 shows the diagram of the developed system. It should be noted that from the designed web user-interface, it is possible to set up the number of Nodes that will be running simultaneously in the system.

The MQTT (message queuing telemetry transport), UDP (user datagram protocol) and TCP protocols were tested to send the samples from each Node. We decided to use TCP as it is the most stable and efficient protocol for the Nodes-to-Server communication. The tests carried out with the MQTT library caused the Nodes to block frequently in long time periods. Another factor for choosing TCP is that it guarantees the delivery of our packets in a sequential manner even if the Nodes are in different subnets. Besides, one additional network layer is avoided by working directly with TCP, since MQTT is above TCP, and in this way some resources are released in the Nodes. As for the tests performed with UDP, it is observed that packets arrive correctly, with a packet loss rate of less than 1%, if the Nodes are in the same network and there is not much traffic. However, as soon as the Wi-Fi traffic increases or different subnets are used for the Node connections, the packet loss rate also increases. Therefore, for our purposes, the best choice is the TCP protocol, because of its low packet loss rate, and the savings in memory and processing resources in the Nodes.

Although TCP guarantees the connection, if there were many retransmissions due to network congestion or due to any Node down, then packet loss could occur and possibly the connection would be restarted (TCP RESET). The RPI Server and Node codes were prepared to detect this situation and report the number of missed packets as soon as the connection is reestablished.

Another critical situation may be the loss of Wi-Fi connection during the sampling process. In this case, the TI libraries in charge of the Wi-Fi connection are programmed to reestablish the connection internally. Besides, control routines were also implemented in the Nodes to reestablish the connection with the RPI Server.

Due to the characteristics of the signal to be sampled (ambient vibrations or seismic noise), the proposed system was designed to work with a sampling frequency of 100 Hz, what it is enough for the expected frequencies. The three components of the seismic sensor were digitized through the 12-bit ADCs incorporated in the microcontroller. A minimum of 2 bytes (16 bits) is required to record each of these data. Besides, for synchronization purposes, a millisecond mark is also transmitted with the data. Thus, a constant rate of 100 samples × 4 values × 16 bit = 6.4 Kbps is the minimum payload that the protocol must assume. To be as efficient as possible, a single Ethernet frame is used to transmit a block of samples. The maximum size of the Ethernet frame is 1518 bytes, so we will transmit blocks of 100 samples (800 bytes) every second, as two seconds would exceed the maximum size and we will have to use segmentation.

### 2.2. Raspberry Pi Server

The server was developed over a Raspberry Pi 3 Model B v1.2 [44] with Raspbian GNU/Linux 9 core 4.19. This model incorporates the Wi-Fi interface that can be configured as an access point (AP) to provide coverage to the nearest Nodes. In our architecture the RPI Server is connected to the internal network through the RJ45 connector and the internal network is responsible for providing connectivity to the different Nodes. This computer was chosen because one of the objectives is to design a portable, low-cost system that allows us to easily instrument a building temporarily. In this sense, the Raspberry Pi computer meets the hardware requirements needed by the developed system, so it is not necessary to add a laptop or a dedicated computer, which would increase the cost.

The Node.js [45] package was installed in order to provide the Web service and execute the JavaScript code of the Server. The main features implemented in the code are:

- TCP connection management.
- Management of received packets and storage of samples.
- Synchronization management.

The SCP (Secure Copy Protocol) service was also installed to access the sample files and download them for a possible subsequent signal processing, for example with MATLAB.

#### 2.2.1. TCP Connections

In order to identify from which Node each of the received packets comes, a TCP port was created for each Node, instead of creating a generic one. Thus, Port 8001 will be used for Node 1, 8002 for Node 2 and so on. The RPI Server will create as many ports as the number of Nodes specified in the configuration and will wait for a new connection through the socket. In this way, it is much easier to identify the origin and to group the samples. When a connection is created, a Hello message should be received to confirm the Node number and the socket will be stored in an array to manage the traffic to be sent.

A timeout was activated in each connection to detect the shutdown of a Node, being 32 s that correspond to the loss of three consecutive Hello messages. Two seconds were added to the timeout for considering any possible delay in the local network due to increased traffic or any other cause.

As already mentioned, in case of network congestion it is very likely that connections will be lost due to excessive TCP retransmissions or Timeout. However, while these connections are being recovered, if the Nodes are in Sampling Mode, they will not be able to send the samples and this is when the loss of samples will occur since the Nodes will not stop sampling. For this reason, it is necessary to detect and report the loss of segments and samples. To do this, the RPI Server keeps track of the number of samples it expects to receive for each Node. If at the reception of a segment these do not match, the RPI Server will calculate how many samples have been lost and increment the corresponding variable. As soon as the Node resets the TCP connection, it will resend the segments containing the samples.

For the connection between the JS Server and the client web browser, WebSockets [46] were used and a series of events was defined, which will be sent as they occur. 

#### 2.2.2. Packets Management and Sample Recording

Several types of packets from our management layer were implemented. As for the packets containing the samples, if there is a web connection, one sample out of 10 contained in the packet will be sent to the client’s web browser in order to not saturate it. This means that for every 100 samples contained in a packet, sample 1, 11, 21... and so on will be sent. To graphically represent these samples in the client’s web browser, the Highcharts [47] object for JavaScript was used.

We implemented the option to save the samples (SAVEDATA) in binary files to be able to process them later (for example in MATLAB). The samples will be saved in files every 15 min that the code itself will generate.

#### 2.2.3. Synchronization Management

RPI Server provides the date/time using the NTP (network time protocol) protocol. For this, an NTP client was configured to obtain the correct date and time from an NTP server, and then an NTP server was configured so that the RPI Server itself provides the date and the time to the Nodes.

The synchronization process between the RPI Server and the Nodes will be detailed later on.

### 2.3. Nodes

The TI CC3200 platform [43] was used for the development of the Nodes due to its low power consumption and the easy integration through its Wi-Fi interface. Specifically, the CC3200 LaunchPad development board was chosen.

The main functions carried out by the Nodes are:

- Controlling the main timer and synchronization;
- Controlling the sampling;
- Controlling the Wi-Fi and NTP connections;
- Controlling TCP connection;
- CLI (command line interface).

**Figure 2:**
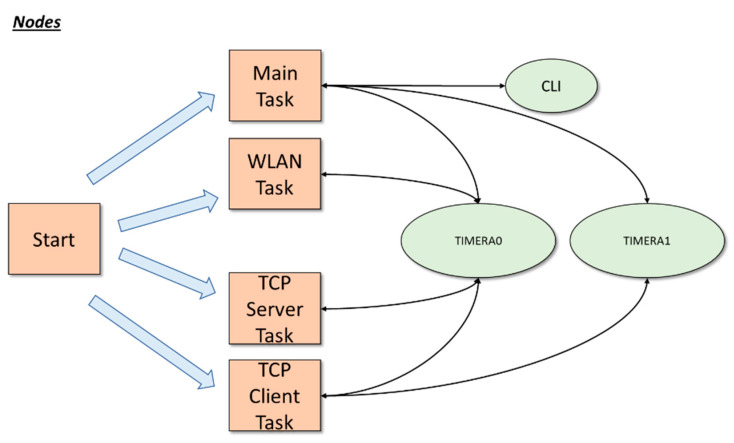
Block diagram of the Nodes software. The main functions (square blocks) and interrupts (oval blocks) are shown and their relationship.

The block diagram used for the Nodes functioning is shown in Figure 2. The messages used between interrupts (*TIMER_A0*, *TIMER_A1* and *CLI*) and tasks (*Main*, *WLAN, TCP Server* and *TCP Client*) are also indicated in the block diagram. 

Three interrupts were enabled:

1. *TIMER_A0.* It is the sampling timer and occurs every 10 milliseconds for capture the samples. By default it is disabled until a START command is received from the RPI Server.
2. *TIMER_A1.* It is the main timer of the program and always occurs every one second.
3. *CLI* (command line interface). It is activated every time that the UART0 receives a character. This interrupt is used to receive user commands through the UART0 and enable logging.

Besides, four tasks are also created in the *Start* module. The tasks in execution are:

1. *Main* task.

It is responsible for executing the main functions and routines. The first step is to read the configuration parameters from a flash memory. After that, main task accesses to an infinite loop where all messages that the main task should handle are. It is also responsible for starting and stopping the sampling process, and for setting the timers to be synchronized with the NTP server. Finally, it is in charge of opening and closing the CLI command line session.

2. *WLAN* task.

In this task, the functionalities associated with Wi-Fi connectivity, time acquisition with an NTP server, and the calculation of the delay and drift time with respect to the RPI Server are carried out.

3. *Server* task.

This task is in charge of monitoring port 800X and waiting for TCP packets to be received. The different frames that were defined are also implemented and processed. This task is related to the receiving part of the communication.

4. *Client* task.

This task is responsible for managing the socket that communicates with the RPI Server and therefore for sending the packets. It is in charge of constructing the frames defined in our protocol and sending them to the RPI Server, including the frame with the samples that have just been collected.

#### 2.3.1. Sampling Mode

Nodes are connected to a three-component sensor through a conditioning circuit [40]. In our case, the sensor Mark-l-4C3D, with a natural frequency of 1 Hz, was used. The conditioning circuit is connected to the three inputs of the ADC1, ADC2 and ADC3 to sample each of the three components. When a Node receives a START-SAMPLING packet, the Timer_A0 is activated and triggers every 10 ms. In the interrupt routine, the first thing that is done is to read the value in milliseconds of the TimeStamp (i.e., MilliTimeStamp, mTS) carried by the Node through Timer_A1 and then the values of the three ADCs are read. These four values (mTS, ADC1, ADC2, ADC3) are stored in a buffer and the counters in charge of controlling the number of total samples (*numtotalsamples*) and the number of samples per packet (*countsamplespkt*) are incremented. When 100 samples are taken, that is, every second, the MSG_SEND_PKT_TCP event is sent to the ClientTask to generate the packet with the 400 values (mTS, ADC1, ADC2, ADC3) and send it to the RPI Server. The inclusion of the mTS information in each packet helps to verify that the sample rate remains stable throughout time. In order to know if the sampling of the signal might generate any type of congestion in the resources of the microcontroller, the duration of the sampling routine was measured. The results show that every 10 ms (sampling period), the routine spends 4 ns in the data acquisition. Therefore, the performance of the other tasks carried out by the Nodes is guaranteed.

#### 2.3.2. CLI Interface

The UART0 was initially enabled as an aid to the development and troubleshooting of the code. A library was developed with a series of commands that allow displaying the status of each Node and the value of the variables used. Besides, the Wi-Fi selection (SSID and password parameters) can be also configured through one of these commands, either for the first time or because a change network is required. 

### 2.4. Wi-Fi–TCP Communication

This is one of the most important blocks in the designed system since it must be capable of transmitting all the captured samples to the RPI Server without any loss. As the CC3200 board incorporates the Wi-Fi interface inside, the sending and receiving of messages can be handled from the designed code using the libraries provided by Texas Instruments.

We chose the TCP protocol as the container for our own layer and packet system. Three tasks were created to manage all the events created: Wi-Fi, ServerTCP and ClientTCP Tasks. 

Once all the peripherals are initialized and the required variables (e.g., the SSID and the password) are read from a flash memory, the Main task starts the Wi-Fi connection by sending an event to the Wi-Fi task. At this moment, the board automatically performs everything necessary to connect to the wireless access point (AP or WAP) and provides an IP. If there were any errors, the function would return an error code. Once the connectivity is established, the current date/time will be requested to the NTP server in order to be synchronized. 

As previously mentioned, the ServerTCP task is in charge of listening and receiving TCP packets coming from the RPI Server, and the ClientTCP task is in charge of creating and sending packets to the RPI Server. 

Nine different types of messages were defined, being identified through type field (byte 2) and code field (byte 3) of each frame (Table 1).

Hello messages are generated on the Nodes every 10 s and serve to notify the RPI Server that the Node and the socket are alive. Two fields TimeStamp (TS) and milliTimeStamp (mTS) are added to each Hello message to tell the RPI Server the time just before sending the packet. The TS (4 bytes) field is the time in the UTC system and the mTS (4 bytes) field corresponds to milliseconds. These messages will be an important part in maintaining the synchronization of the Nodes with respect to the RPI Server.

**Figure 3:**
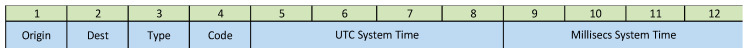
Structure of the Hello message.

The structure of the Hello messages is shown in Figure 3. It is composed of the following fields:

- Fields 1 and 2 (Origin, Destination). Indicates who is sending the message and who receives it.
Master  ➔ 0Nodes  ➔ 1, 2, 3 …
- Field 3 (Type). Indicates the type of message.
- Field 4 (Code = 0).
- Field 5 (TimeStamp). System Time in UTC seconds
- Fields 6. (milliTimeStamp). Milliseconds System Time

Type 1 messages (SO, Sampling Order) are used to order the START/STOP of the sampling according to the Code value. The possible orders are:

- Code = 1: Indicates that the Node starts sampling immediately by sending the samples.
- Code = 2: Indicates that the Node immediately stops sampling and sends the pending samples.
- Code = 3: Indicates that the Node starts sampling immediately without sending the samples. This option is for when the time and drift of the Node clock need to be calibrated.
- Code = 5: Indicates that the Node starts sampling at the next second and start sending the samples.

**Figure 4:**
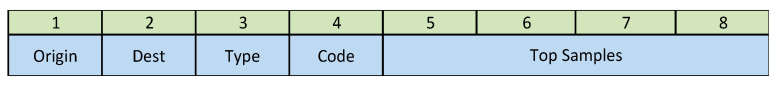
Structure of the *Sampling Order* message.

The structure of these types of messages is shown in Figure 4.

**Figure 5:**
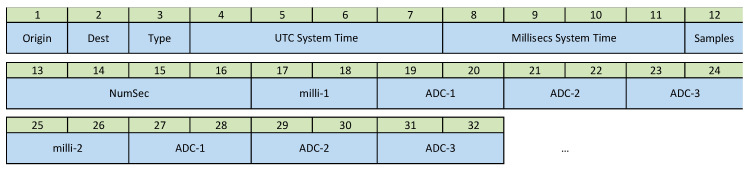
Structure of the *Samples Reply* message.

The Type 2 messages (Samples Reply) are the packets containing the data included in the 100 samples. Concretely, this information corresponds to the three ADC channels and the mTS variable, making a total of 400 values (800 bytes). Taking into account that the header occupies 16 bytes, the maximum size of these messages will be 816 bytes. A message will be generated every second. The Samples field indicates the number of time slots contained in the message (1–100) and the NumSec field indicates the sequence number of the packet which will be the number of the first sample of the message in the total computation. The structure is indicated in Figure 5.

**Figure 6:**
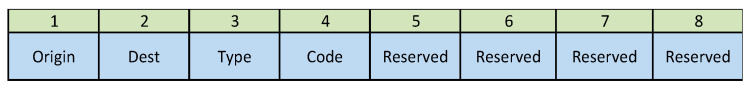
Structure of the *TimeStamp* message.

Type 7 messages (TimeStamps) are the packets used to indicate to the RPI Server the precise instant at which the Node starts sampling the first sample of the configured block (Code = 1), the instant at which it samples the sample indicated in the Stop-and-Go process (Code = 2), or the instant at which it finishes the last sample of the configured block (Code = 3). These messages, once received by the RPI Server, will serve as a time reference to calculate the duration time of a configured sample block by simply calculating the time difference between a Code1 and a Code3 message. In Figure 6, the structure of these types of messages is shown.

The remaining messages are related to timing and synchronization and will be detailed in Section 2.5.

**Figure 7:**
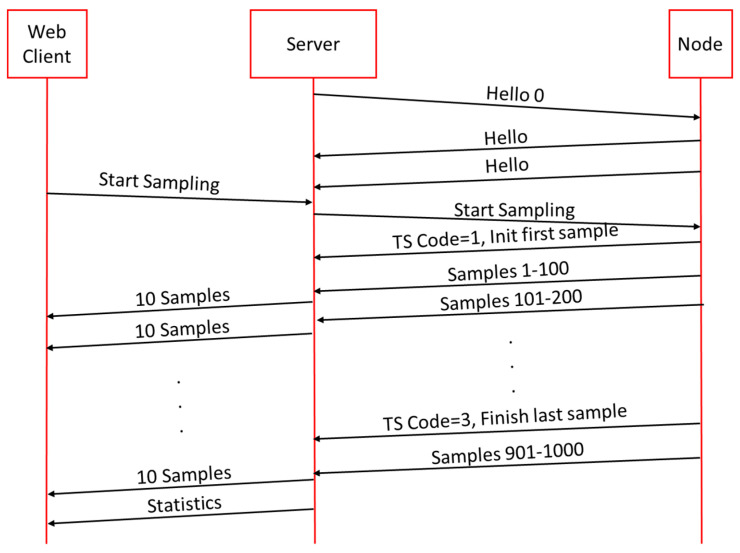
Sequence of one block of 1000 samples.

The sequence of messages shown in Figure 7 is an example for a block of 1000 samples, so the duration will be 10 s. When the Nodes are not sampling, they are sending Hello frames every 10 s to indicate that they are alive. When the user sends the START command through the web browser, the TopSamples field will indicate the number of samples, that is 1000 samples in this example. This mode is called Limit Mode. If, on the other hand, no number of samples is specified, this mode is called Continuous Mode and the Node will never stop sampling until it receives a STOP command. When the Node receives the Start Sampling frame, the process starts and just when it is going to read the ADC1 value, the Code = 1 frame (message type 7) is sent so that the RPI Server registers the time T0, which will be the sampling start time. Then the Node will send the stored samples in batches of 100 samples. When the Node finishes reading the 1000th sample, then it will send a Code = 3 frame and the RPI Server will register the time when it received this frame. Then the Node will send the last packet with the remaining samples to be sent and end the sampling. When the RPI Server receives the last packet with the last samples, it will calculate the statistics of times, packet loss, etc., and these statistics will be sent to the Web Client for visualization.

As long as there is a Web Client connection, the RPI Server will send a set of 10 samples to the client to be drawn in the HighChart object. Each time the RPI Server receives a packet with the 400 values (mTS, ADC1, ADC2, ADC3), it will extract the four values of the time intervals 1, 11, 21, 31... until it has the 10 and will send them through a WebSocket to the Client browser where it will draw the shape of the signals interpolating the 10 sent samples. The purpose of plotting the signal is to provide an estimate of the detected signal and not the signal in detail as it would consume many resources in the web browser because Node.js is very heavy. Tests were carried out sending a larger number of samples but from 20 samples onwards the web browser will produce delays and stops in the visualization, even slowness in the interaction with the controls, which produce crashes and a poor response from the web browser. 

When the system is registering, each Node generates a constant traffic of 6.5 Kbps, so we must consider the load of the network so that no data is lost. The theoretical limit of our system in terms of the maximum number of Nodes that can be supported will be given by the load of the network and the RPI Server. If the network interface of the RPI Server runs at a theoretical velocity of 1 Gbps, considering a limit of 25% so as not to saturate the OS, RPI processes and the Node.js server, we get an approximate theoretical calculation of 38 Nodes. In the experiments we tested up to two Nodes working perfectly.

### 2.5. Synchronization Process

A new synchronization scheme was designed based on the Precision Time Protocol (PTP) [48] and the Doze Mechanism [49]. The developed approach consists of two phases: the first phase calculates the offset and drift between the Nodes and the RPI Server; the second phase tries to correct the Node time drift.

For the synchronization process of the Nodes, new messages types 4, 6 and 9 were created.

The Nodes use the timers provided by the hardware of the CC3200 board and its quartz crystal. These crystals are not of very good quality and therefore small timing differences occur, which are increased over time (drift). In order for the Nodes not to have time differences at the moment they have to read the samples (jitter) and to be as accurate as possible when taking the sample, they must have the same time reference as the RPI Server. Thus, the offset and drift of each Node with respect to the RPI Server must be calculated and its time reference adjusted.

**Figure 8:**
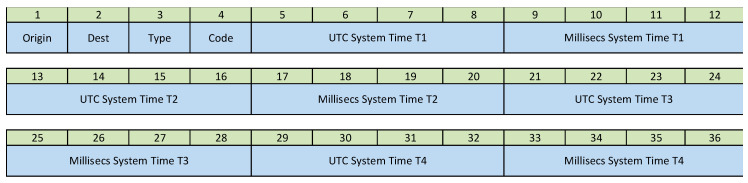
Structure of the synchronization (SYNC CLK) messages.

The calculation of the offset and drift values is based on the operation of the PTP protocol but with modifications. The synchronization process is managed by the RPI Server that will initiate the exchange of messages between RPI Server and Nodes. Figure 8 shows a part of the message sequence.

**Figure 9:**
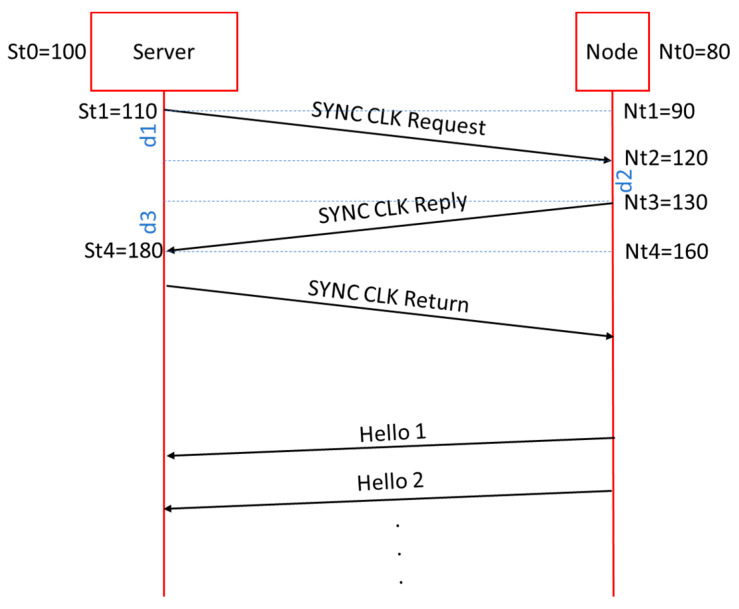
Sequence of synchronization between RPI Server and one Node.

In type 9 messages, the TimeStamps of the time of sending each phase are added to the frames until the four TimeStamps required for the calculations are completed. These four fields are the TimeStamp value in UTC format and in milliseconds. Each of them occupies 4 bytes. The third type of message, SYNC CLK Return, is added to return the complete time sequence to the Node so that it can perform the calculations by itself and thus be able to adjust the local time. The delay and time drift respecting the RPI Server time are calculated by equation [1], the meaning of whose variables are in Figure 9. For the example shown in the Figure 9, it gives us values of delayT = 60 ms and drift = −20 ms, that means that the Node has a delay of 20 ms so it will have to advance its Timer about 20 ms.

(1) $$T_{T}=d_{1}+d_{2}+d_{3}\;delay_{T}=\left(St_{4}-St_{1}\right)-\left(Nt_{3}-Nt_{2}\right)\;drift_{T}=\left(Nt_{3}+\frac{d_{T}}{2}\right)-St_{4}$$

Since the transmission times of messages in a Wi-Fi network are inconsistent and when the higher the network traffic the worse are the times obtained, it is necessary to implement methods that provide stability and a better approximation to the values of the calculated times. For this purpose, an algorithm based on the Doze Mechanism [49] was implemented, which consists of two phases. 

#### 2.5.1. Synchronization, Phase 1

**Figure 10:**
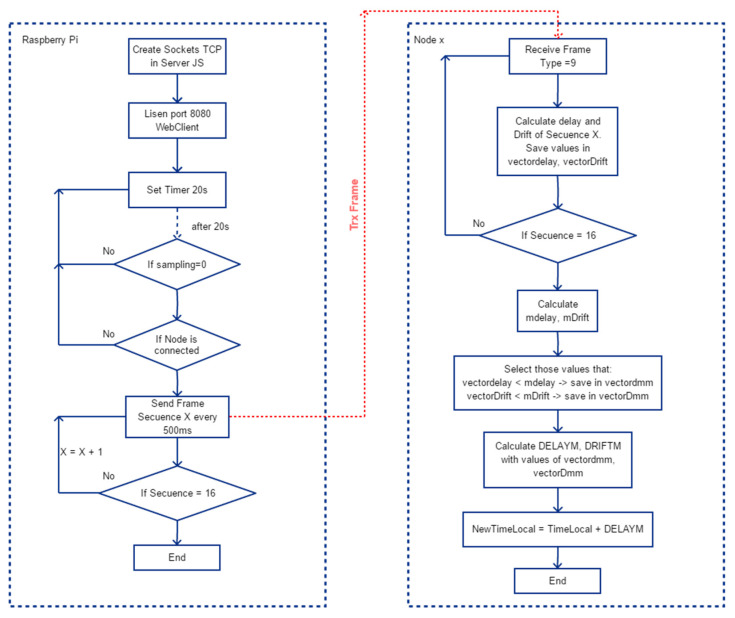
General flowchart of the delay and drift algorithm in phase 1.

After 20 s when a connection is established between the RPI Server and a Node, a sequence of 16 SYNC CLK Request-Reply-Return messages is started and all the TimeStams of the messages are stored in a buffer. Then the 16 values of delay and drift are calculated and the average of these values, mdelay and mDrift, is obtained. Then the delay and drift values below the corresponding average are selected and stored in the buffers vectordmm and vectorDmm, the rest are discarding the other values. The average of the selected delay and drift values is recalculated obtaining DELAYM and DRIFTM. Finally, the local clock reference is adjusted according to DELAYM and DRIFTM. With this method, those messages that for some reason have generated greater transmission delays and that can alter the calculated averages are discarded. In Figure 10, the flowchart of the algorithm phase 1 is shown.

#### 2.5.2. Synchronization, Phase 2

In phase two we try to keep the time reference synchronized with the RPI Server. For this, Hello messages are sent by the Nodes every 10 s, containing the TimeStamp. The RPI Server will collect and store the 16 TimeStamp values of the received Hello messages together with the reception time and will recalculate the DRIFTM value as it is done in phase 1. If DRIFTM is greater than 20, it will mean that it has an offset of 20 ms and therefore the local clock will be reset. Otherwise, it will continue calculating a new DRIFTM value for each Hello received. At the time of a local clock reset, 16 new Hello messages must be stored, which will take 160 s. This will be done while in Waiting Mode, i.e., without sampling.

In phase 2, it is differed whether the Node part is in Sampling Mode or not. If it is in Sampling Mode and a Type = 6 frame is received, it means that we have a sample offset with respect to the other Nodes. If the Node is ahead, it means that the number of samples it is counting (numsample) and doing is higher than that of the other Nodes and therefore we will have to subtract that number of samples from the rest, but if on the contrary we are behind then we will have to advance the numsample counter. This action will imply that in the buffer where the samples are stored there will be a jump and therefore the positions that are skipped decided to remain with the value zero. It could have been decided to fill it with the value of the last sample taken but it was decided to zero in order to detect the synchronization instants.

**Figure 11:**
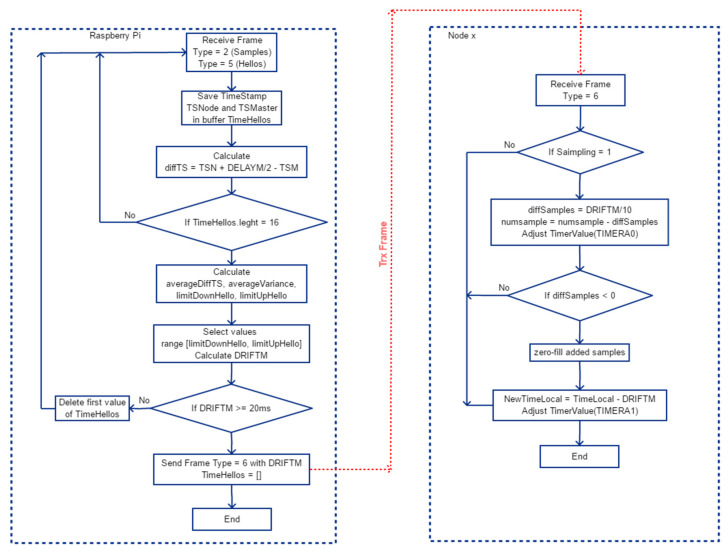
General flowchart of the drift algorithm in phase 2.

In Figure 11, the flowchart of the algorithm phase 2 is shown.

**Figure 12:**
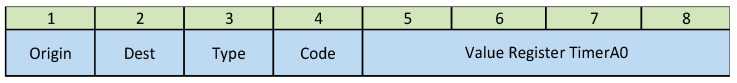
Structure of the Set Timer, Sampling Timer_A0.

Note that to have a first fine tuning of the local Timer, a calibration must be performed for 12 or 24 h to calculate the value of the Timer_A0 Counter Register and send it to each Node with the message Set Timer (Type = 6, Code = 1). Even so, the effect of drift will remain, but minimized. Figure 12 shows the structure of the message.

The Nodes have a main clock frequency of 80 MHz and to get the sampling interrupt to trigger every 10 ms, the Timer_A0 Counter register should be 800,000. In the experimentation section we will see how the Nodes do not have the same quartz crystal frequency.

For the Sampling Mode, two methods were implemented for the maintenance of the synchronization of the Node: Stop-and-Go and Synchro. Both can be activated or deactivated from the Web client.

#### 2.5.3. Stop-and-Go Method

This is the first method implemented to correct the effect of time drift. In Sampling Mode, it was detected that as time progressed, although the local clocks of each Node were adjusted, a small deviation (drift) was accumulating. To readjust the sampling time intervals in all Nodes, this method was implemented. It consists of the Nodes themselves stopping sampling at a certain number of samples defined in the Set Stop-and-Go message (Type = 4) and when the RPI Server has received the message containing the last samples from all the Nodes then immediately send the order to continue to the next block of samples. This will cause the fastest Node to wait until the slowest Node has sent its samples to the RPI Server, restarting all of them at the same time.

#### 2.5.4. Synchro Method

In this mode the Nodes are continuously synchronized as the DRIFTM exceeds the threshold above 20 ms. It is the same method as phase 2 but using the TimeStamp field that incorporates the sample messages (Type = 2) that are received every second. In this case the algorithm waits to store 16 TimeStamp values, which will take 16 secs, and then starts calculating the DRIFTM in the same way as in phase 2, selecting the best values, and at each reception of a type 2 message containing the samples. If the calculated DRIFTM exceeds 20 ms, that is two samples of deviation, then a Set Timer-Delay Timer message (Type = 6, Code = 5) containing the milliseconds of difference (DRIFTM) is sent from the Server. When the Node receives the message, it replies with the Set Timer-Ack message (Type = 6, Code = 1) and immediately adjusts its Sampling Timer according to the value passed. The structure of the Set Timer messages is the same as that shown in Figure 10.

### 2.6. Save File System

In the web user-interface options of the Web client, we added the option to save the samples received in the RPI Server and then download the files with an SCP client. These files are automatically saved with the name “SAMPLES_DATE_TIME” where DATE is the date and TIME is the time of creation of the new file. A file will be generated every 15 min due to the size of the files. The content of the files is exactly the messages Type = 2, in binary format to reduce the file size. Concretely, the size of these files will be 1435 KB for a 15 min recording.

There is also the option to create and save the Debug messages in text mode for later analysis in case of failures.

### 2.7. User Interface of the Web Client

**Figure 13:**
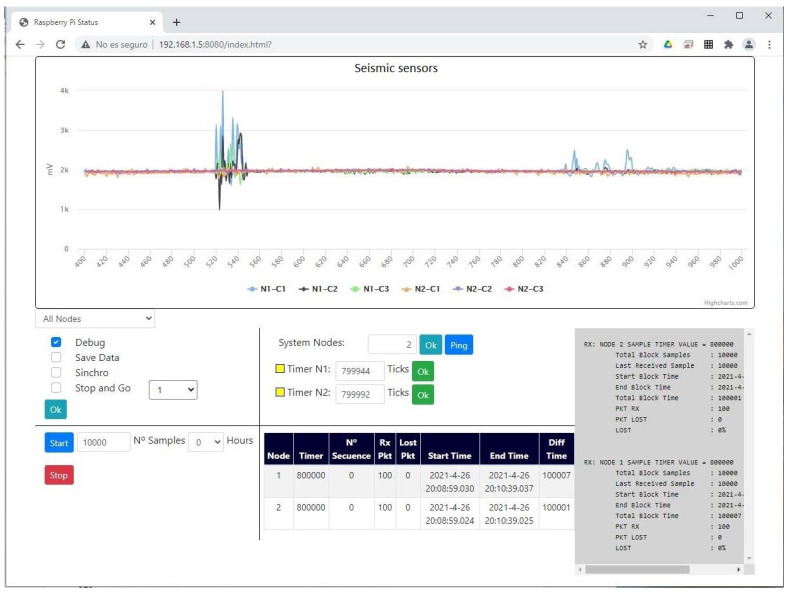
User interface of the Web client.

The Web client part (Figure 13) was also developed with JavaScript language. It consists of a panel where the current signal is displayed, taking into account that only one of every 10 samples received is displayed so as not to saturate the RPI Server or the Web client. It is to display an approximation of the received signal. We can select to display only the signal of a particular Node or a particular component. Another panel is the Options panel where we find Debug, SaveData, Synchro and Stop-and-Go.

From the web page, the number of Nodes can be configured, generating the objects required for each Node. A panel was also designed to display a table with the important data related to the current sampling block such as the start of the sampling, the number of packets received, etc. Additionally, on the right side of the screen a panel was implemented to display important system messages and configuration, as well as the statistics at the end of a sampling block.

## 3. Results and Discussions

### 3.1. Technical Characteristics of the Designed Prototype

As a result of the present investigation, a seismic data acquisition prototype was implemented with the following technical characteristics:

- Nodes:
  - Power supply of 5 V, obtained from batteries or microUSB charger.
  - Model used: LaunchPad CC3200.
  - Serial CLI interface at 115200 bauds for monitoring and provisioning tasks.
  - Connected to three-component sensor (e.g., 1-Hz Mark-L-4C3D).
  - Consumption:
    ▪ 70 mA Mode Standby;
    ▪ 80 mA Mode Sampling;
    ▪ 140 mA pp on start.
- Raspberry Pi Server:
  - Power supply through the microUSB charger.
  - Model used: Raspberry Pi 3 Model B v1.2 [44].
  - Location: In a network outlet.
  - Installed services: SSH, NTP Server, NTP Client, SCP, Java, Apache, Node.js.
  - Consumption:
    ▪ 320 mA normal operation;
    ▪ 450 mA pp on start.

**Figure 14:**
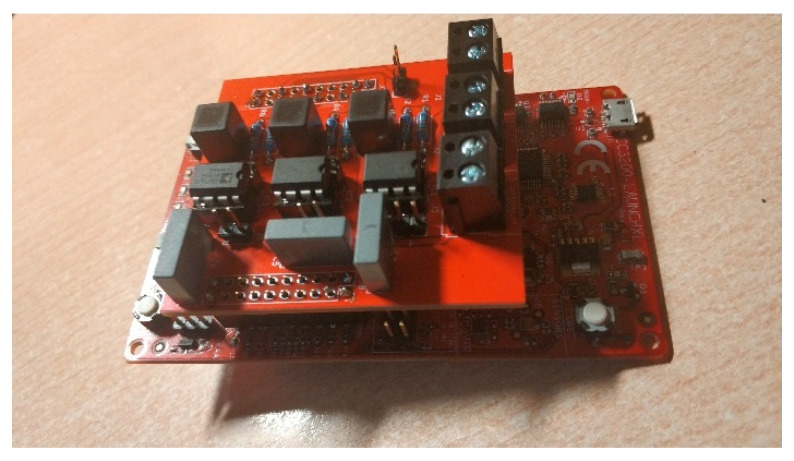
Conditioning circuit assembled into Lanchpad CC3200.

Figure 14 shows the prototype of the conditioning circuit connected to the LaunchPad CC3200 backplane. The black connectors for the connection with the three-component sensor are shown.

The cost of each part of the installed system is detailed below:

- Nodes:
  - LaunchPad CC3200: EUR 55.44.
  - Conditioning circuit: EUR 43.51.
- RPI Server:
  - Raspberry Pi Kit: EUR 101.33.

For the experimentation part, two Nodes were used with a total cost of EUR 200.28.

### 3.2. Experiments

In order to test the correct functioning of the synchronization and sampling processes, a series of tests were performed in a two-floor house with Wi-Fi connectivity. The scenario developed is as follows: two Nodes were installed, one on the first floor and the other on the opposite side of the second floor. Each of them connected to different AP and within the same LAN together with the RPI Server. The same output of the sinusoidal signal generator (Multicomp MP750064) was connected to each Node’s ADC1, which generates a 1 Hz and 1.2 Vp signal, so both Nodes receive the same signal at the same time. Therefore, the signal obtained from the user interface of the Web client should be exactly the same.

The first thing to do is to perform a calibration to determine the appropriate value for the counter register of Timer_A0, which is in charge of controlling the 10 ms sampling interval. 

**Figure 15:**
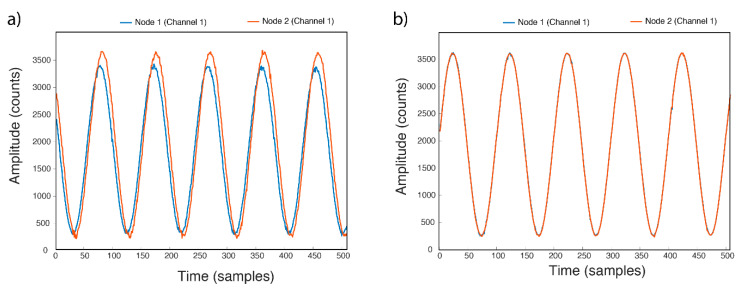
Start delay between Nodes: (**a**) without synchronized start, (**b**) with synchronized start.

The initial test is related with starting synchronization. At the beginning of the sampling process, the Nodes start at different times and there are delays between 20 and 80 ms from the beginning. It is due to the fact that the transmission times of the packets in the network are different because some Nodes are further away from the RPI Server than others. To ensure that all Nodes start at the same instant, each Node was configured to start sampling at the beginning of the next second, that is when Timer_A0 triggers its interrupt, when MilliTimeStamp is 000. If the Nodes are completely synchronized using the implemented techniques, that means that the Nodes have almost the same time and their Timers_A0 trigger almost in unison, and therefore they will start almost at the same time. This was designed to avoid delays or differences in the sampling times between the Nodes, or at least that they are as small as possible. Figure 15 shows the initial phase shift of the same signal (1 Hz sinusoidal signal) at the two Nodes.

After that, the time drift and the permanency of the synchronization were analyzed. The result of the 24 h register is shown in Table 2 where the time difference between the two Nodes for the same block of samples (24 h) can be observed. Node 1 (5048 ms of excess) samples slower than Node 2 (4292 ms of excess) and in turn both take longer than the stipulated time.

With these results the calculations for the new values for the counter register of Timer_A0 give us the following:

- Nodo 1: counter_reg = 799,951;
- Nodo 2: counter_reg = 799,965.

The results obtained from a new 24 h register is shown in Table 3.

With these new values, the sampling frequency does adjust more accurately to 100 Hz.

**Figure 16:**
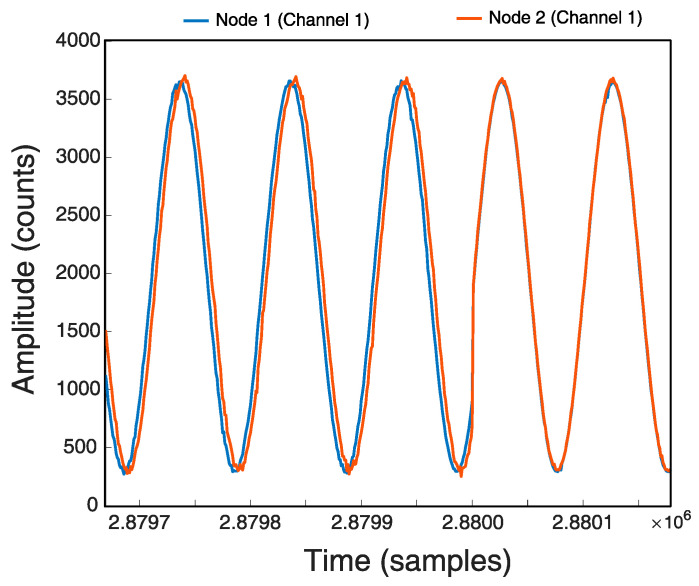
Sincronization with Stop-and-Go.

Figure 16 shows how the two Nodes are synchronized when they reach sample 2,880,000, i.e., 8 h after starting, with the Stop-and-Go method. The next synchronization will take place after 8 h, i.e., at sample 5,760,000.

If we look at the test log in Table 4 we can see how Node 2 reaches the sample 2,880,000 at the 962 ms instant and Node 1 does it at the 54 ms instant of next second, so there is a 92 ms lag. Then we see how the two Nodes send the ACK almost at the same time (56 and 58 ms) meaning that they sample almost at the same time.

**Figure 17:**
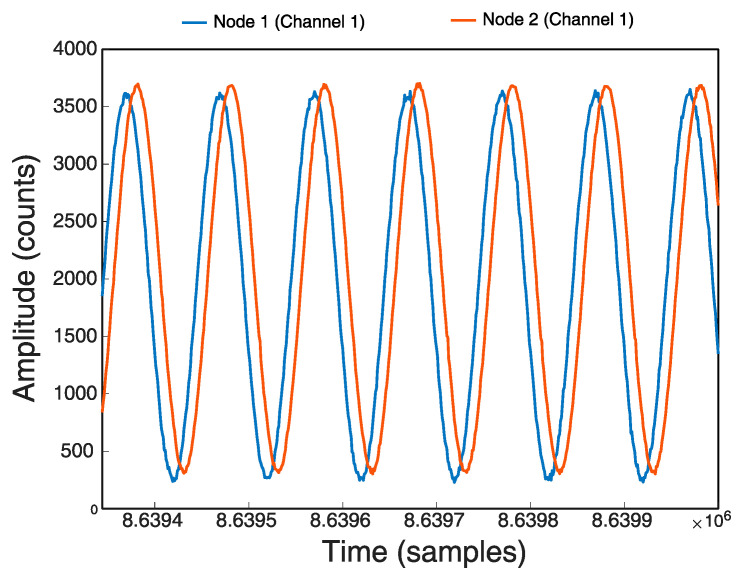
Final signal in 24 h register with Stop-and-Go.

The final result of the 24 h recording with the Stop-and-Go method configured at 8 h is shown in Table 5, which shows a time lag of 93 ms for Node1 and 83 ms for Node2 with respect to the time it should have taken. The time lag between them is only 10 ms. Figure 17 shows the signal at the end of the recording observing a deviation with the Stop-and-Go method.

As for the second implemented method, Sinhcro, the test results show a better synchronization since the adjustments are more continuous and do not drag the drift in time.

**Figure 18:**
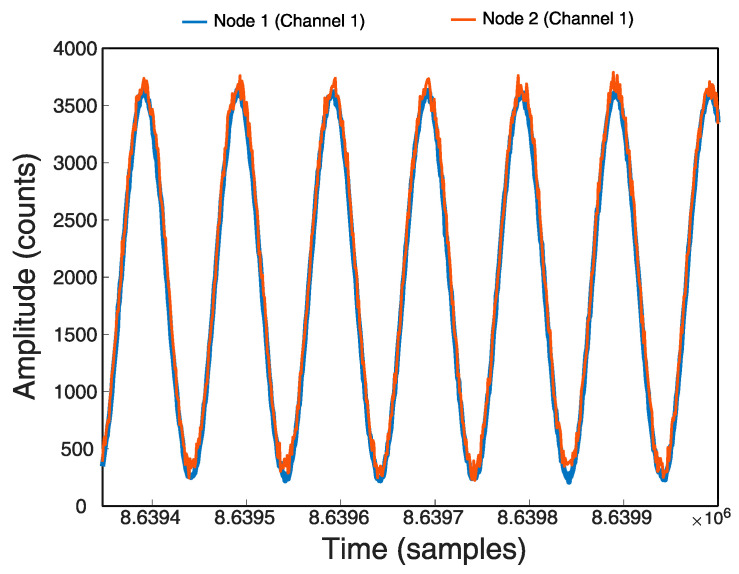
Final signal in Register 24 h with Sinchro.

Table 6 shows the final result of the 24 h recording with the Sinchro method. The times improved by 78% for Node 1 (71 ms) and almost 65% for Node 2 (84 ms), the signal difference between the two Nodes is 13 ms and can be seen in Figure 18. As seen in the log, Node 1 was reset about seven times and Node 2 about 14 times.

The comparison of the signals recorded in each of the Nodes shows a correct synchronization, especially when the developed Sinchro method is used.

In the tests carried out, there were periods of time in which network congestion occurred. This situation caused packet loss. The observed effects were delayed in the delivery of packets and even momentary loss of connection with the Nodes. When packets are delayed due to congestion, this directly affects the instantaneous calculation of the DELAYM and TimeStamps fields. This causes the RPI Server to believe that the Node time has been mismatched and sends the order to delay or advance the sampling instant depending on whether the difference is positive or negative. This action will cause the Node to become misaligned with respect to the others and will result in loss of samples in the case of a delay command. Delay mismatches will occur more frequently when congestion occurs. As for the situation of connection loss, while restarting, those samples captured by the affected Node will be lost due to the impossibility of being able to send them. For this reason, the LOST PKT field is indicated in the final results of the log, so that the user can take it into account and can detect such periods by viewing the generated log. The Wi-Fi network is the most susceptible to congestion because it is a shared medium and the channel bandwidth is much lower than that provided by copper or fiber cabling, so it is necessary to prevent the APs from being saturated.

**Figure 19:**
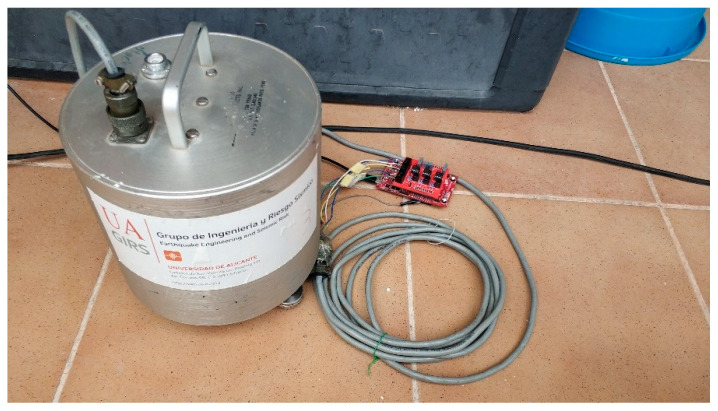
Node 2 with sensor at second floor.

Once we confirmed with the signal generator that the Nodes remain synchronized at all times and maintain the sampling frequency at 100 Hz, we connected the Nodes to the three-component sensors. Figure 19 shows Node 2 connected to the sensor located outside on the second floor.

**Figure 20:**
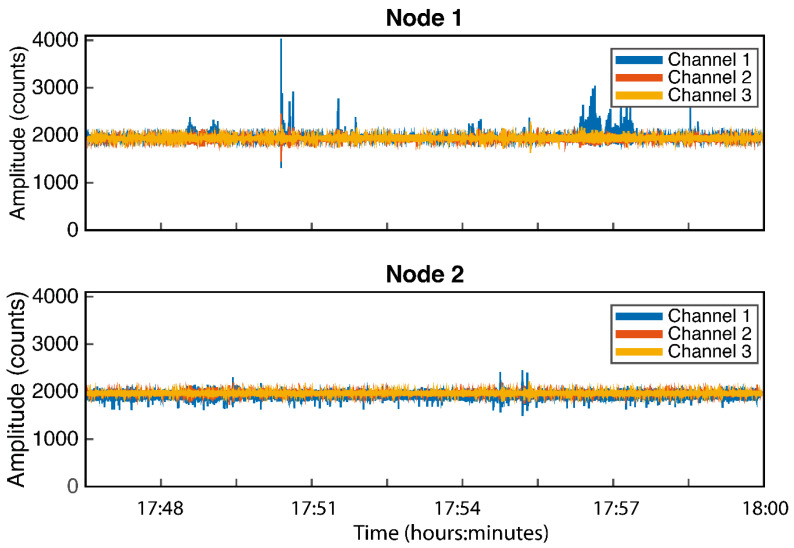
Example of 15 min.

Figure 20 shows in MATLAB the detailed sampled signals contained in one of the files generated by the RPI Server and with a duration of 15 min. The file corresponds to 23 April 2021 between 17:45 and 18:00 h. The peak recorded by Node 1, located on the ground level, corresponds to the opening of a motorized door and the subsequent footsteps of people.

**Figure 21:**
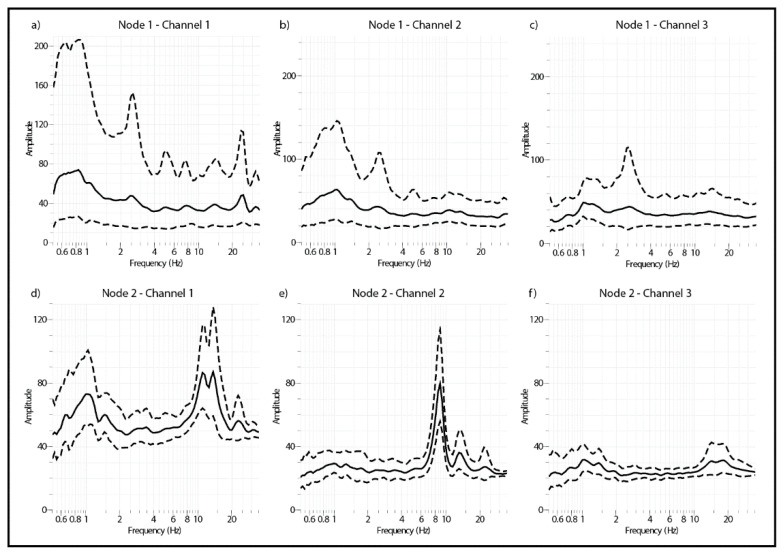
Power spectral density of the recordings shown in Figure 20.

Figure 21 shows the power spectral density of the two Nodes monitoring the building (Figure 20): Node 1 located in the ground floor; Node 2 located in the second floor. Channel 1 of the two Nodes corresponds to the Horizontal-Longitudinal component; channel 2 to the Horizontal-Transverse component; channel 3 corresponds to the Vertical component. The Horizontal-Transverse component of Node 2 clearly shows a peak at 9.03 Hz corresponding to the resonance frequency of the building. According to the study of Vidal et al. [50] (p. 8), the relationship between the period of the building (T) and the number of floors (N) is fulfilled, being for our particular case of 0.11 s (T = 0.054*N), corresponding to 9.09 Hz as reference value of Vidal et al. [50].

Finally, the accuracy of the acquisition system was analyzed. For that, two continuous signals of 700 and 1400 mV (value close to the maximum ADC voltage) were connected to the three input channels and five 30-s recordings were undertaken for each voltage. The average value of each series of records and the corresponding number of counts by µV were calculated and compared with the theoretical value. In Table 7, the obtained results are shown. The system presents a maximum deviation of 0.70 and 1.35% for the half and full dynamic range of the ADC, respectively.

## 4. Conclusions

In this work, a wireless seismic data acquisition prototype was developed. The designed system transmits the signal registered by the Nodes to a central server (RPI Server) and allows displaying remotely the seismic signal in different web browsers at the same time, all in real time. In the developed system, the RPI Server was programmed with Node.js technology that receives the frames with the samples and saves them in files or sends them to the client web browsers. In the developed web user interface, different controls and objects were implemented to start and stop a log as well as different options. Therefore, it is not necessary to have a person in the field to start/stop the capture of the samples. From any place with Internet connectivity, it is possible to send the commands and also to visualize the signals. Besides, it is also possible to download the log files for further processing with a maximum waiting time of less than 15 min since the server generates a file every 15 min.

Wi-Fi connectivity was used in the Nodes to be able to transmit the captured samples in a reliable and orderly way, so it is necessary to have a Wi-Fi network deployed in the working environment. The microcontroller chosen was the LaunchPad CC3200 as it implements a chip to provide the Wi-Fi interface, which also has a 32-bit ARM chip.

The experiments were carried out in a two-story building with two Nodes and it was found that the system is able to synchronize and adjust itself in time automatically in order to correct delays and drifts. 

The use of low power consumption and low-cost components was taken into account in the development. The software and the programming of the different elements were developed with open-source tools.

## Figures and Tables

**Table 1:** Types of messages.

Types of Messages
Echo Reply
Sampling Order, START immediately
Sampling Order, STOP
Sampling Order, START without sending
Sampling Order, START in next sec
Samples Reply
Set Stop-and-Go
Hello
Set Timer, set Sampling Timer counter value
Set Timer, Ack
Set Timer, set Delay Timer value in milisecs
TimeStamp, init sampling
TimeStamp, Stop-and-Go
TimeStamp, finish sampling
Echo Request
SYNC CLK Request
SYNC CLK Reply
SYNC CLK Return values

**Table 2:** Results of 24h register calibration with default values.

RX: NODE 2 SAMPLE TIMER VALUE = 800,000 Total Block Samples: 8640,000 Last Received Sample: 8640,000 Start Block Time: 2021-1-20 19:58:26.219 End Block Time: 2021-1-21 19:58:30.511 Total Block Time: 86,404,292 ms PKT RX: 86,400 PKT LOST: 0 LOST: 0%
RX: NODE 1 SAMPLE TIMER VALUE = 800,000 Total Block Samples: 8640,000 Last Received Sample: 8640,000 Start Block Time: 2021-1-20 19:58:26.212 End Block Time: 2021-1-21 19:58:31.260 Total Block Time: 8,6405,048 ms PKT RX: 86,400 PKT LOST: 0 LOST: 0%

**Table 3:** Results of 24 h register calibration with new values of the Timer_A0 counter.

RX: NODE 1 SAMPLE TIMER VALUE = 799,951 Total Block Samples: 8,640,000 Last Received Sample: 8,640,000 Start Block Time: 2021-3-31 11:08:23.041 End Block Time: 2021-4-1 11:08:22.998 Total Block Time: 86,399,957 ms PKT RX: 86,400 PKT LOST: 0 LOST: 0%
RX: NODE 2 SAMPLE TIMER VALUE = 799,965 Total Block Samples: 8,640,000 Last Received Sample: 8,640,000 Start Block Time: 2021-3-31 11:08:23.024 End Block Time: 2021-4-1 11:08:23.101 Total Block Time: 86,400,077 ms PKT RX: 86,400 PKT LOST: 0 LOST: 0%

**Table 4:** Log of Stop-and-Go occurs.

2021-04-02, 06:09:16.962, STOP AND GO Node-2 Sample = 2,880,000, New value = 5,760,000 2021-04-02, 06:09:17.054 RX(816) -> 1:0:2:1617336557038:2879901-2880000 2021-04-02, 06:09:17.054, STOP AND GO Node-1 Sample = 2,880,000, New value = 5,760,000 2021-04-02, 06:09:17.056 Send START to Node 1 2021-04-02, 06:09:17.058 Send START to Node 2 2021-04-02, 06:09:17.069 -> Receive ACK STOP AND GO code = 2—Node 2 2021-04-02, 06:09:17.135 -> Receive ACK STOP AND GO code = 2—Node 1

**Table 5:** Results of 24 h with Stop-and-Go = 8 h.

RX: NODE 2 SAMPLE TIMER VALUE = 799,964 Total Block Samples: 8,630,000 Last Received Sample: 8,640,000 Start Block Time: 2021-4-1 22:09:17.017 End Block Time: 2021-4-2 22:09:17.256 Total Block Time: 86400239 ms PKT RX: 86400 PKT LOST: 0 LOST: 0%
RX: NODE 1 SAMPLE TIMER VALUE = 799,951 Total Block Samples: 8,640,000 Last Received Sample: 8,640,000 Start Block Time: 2021-4-1 22:09:17.041 End Block Time: 2021-4-2 22:09:17.373 Total Block Time: 86,400,332 ms PKT RX: 86,400 PKT LOST: 0 LOST: 0%

**Table 6:** Results of 12 h with Sinchro.

RX: NODE 2 SAMPLE TIMER VALUE = 799,964 Total Block Samples: 8,640,000 Last Received Sample: 8,640,000 Start Block Time: 2021-4-2 23:41:41.023 End Block Time: 2021-4-3 23:41:41.107 Total Block Time: 86,400,084 ms PKT RX: 86,400 PKT LOST: 0 LOST: 0%
RX: NODE 1 SAMPLE TIMER VALUE = 799,951 Total Block Samples: 8,640,000 Last Received Sample: 8,640,000 Start Block Time: 2021-4-2 23:41:41.037 End Block Time: 2021-4-3 23:41:41.108 Total Block Time: 86,400,071 ms PKT RX: 86,400 PKT LOST: 0 LOST: 0%

**Table 7:** Experimental accuracy results of the data acquisition system.

Input DC signal (mV)	700	1400
Sampling rate (Hz)	100	100
Dynamic range (bits)	12	12
Theoretical value of one count (µV/counts)	358.15	358.15
NS channel (µV/counts)	360.70	363.00
NS deviation from theoretical value (%)	0.70	1.35
EW channel µV (µV/counts)	360.61	362.94
EW deviation from theoretical value (%)	0.68	1.34
Z channel µV (µV/counts)	360.57	362.94
Z deviation from theoretical value (%)	0.67	1.34

## Data Availability

Not applicable.

## Abbreviations

It describes the abbreviations and variables used throughout this paper.

**Abbreviations**	**Description**
SNR	Signal noise ratio
WSN	Wireless sensor networks
MQTT	Message queuing telemetry transport
UDP	User datagram protocol
TCP	Transmission control protocol
RPI	Raspberry Pi
SCP	Secure copy protocol
NTP	Network time protocol
CLI	Command line interface
UART	Universal asynchronous receiver-transmitter
ADC	Analogue to digital converter
WAP or AP	Wireless access point
UTC	Universal time coordinated
mTS	Milliseconds time stamp
TS	Time stamp
PTP	Precision time protocol
SSH	Secure shell
LAN	Local area network
**Variables**	**Description**
numtotalsamples	Number of total samples in actual block register
countsamplespkt	Number of samples included in frame to send
mdelay	Average delay of 16 calculated values
mDrift	Average drift of 16 calculated values
vectordmm	Buffer with those values less than *mdelay*
vectorDmm	Buffer with those values less than *mDrift*
DELAYM	Average delay of the selected values
DRIFTM	Average drift of the selected values
